# Pregnancy and delivery in an advanced cancer survivor with immune checkpoint inhibitor-induced type 1 diabetes: a case report

**DOI:** 10.1007/s12020-024-03780-w

**Published:** 2024-03-19

**Authors:** Keiji Sugai, Takashi Miwa, Junya Kojima, Yuri Ueda, Kiyoaki Tsukahara, Hirotaka Nishi, Ryo Suzuki

**Affiliations:** 1 Department of Diabetes, Metabolism and Endocrinology, Tokyo Medical University, Tokyo, Japan; 2 Department of Obstetrics and Gynecology, Tokyo Medical University, Tokyo, Japan; 3 Department of Otorhinolaryngology, Head and Neck Surgery, Tokyo Medical University, Tokyo, Japan

**Keywords:** Immune checkpoint inhibitor-induced type 1 diabetes, Immune-related adverse events, Pregestational diabetes mellitus, Sensor augmented pump

## Abstract

**Purpose:**

Given the rarity and elderly onset of immune checkpoint inhibitor (ICI)-induced type 1 diabetes (ICI-T1DM), cases leading to delivery are rare.

**Method:**

To our knowledge, this is the first case report of childbirth in a patient with ICI-T1DM after cancer survival. A 32-year-old woman was started on Nivolumab for metastatic parotid cancers one year after total parotidectomy.

**Result:**

The patient developed ICI-T1DM after 43 cycles and started multiple daily insulin therapy and self-monitoring of blood glucose. Complete response was maintained for 2 years by nivolumab, and she finished nivolumab in 77 cycles to attempt pregnancy. During the follow-up period, she began using a sensor-augmented pump (SAP). She had undetectable serum and urinary C-peptide when she started SAP. Her HbA1c level decreased from 7.8 to 6.6% without increasing hypoglycemia in one year. The patient remained in complete response after ICI discontinuation, and embryo transfer was initiated. Pregnancy was confirmed after a second embryo transfer (21 months after ICI discontinuation). At 36 weeks and 6 days, an emergency cesarean section was performed due to the onset of preeclampsia. The baby had hypospadias and bifid scrotum but no other complications or neonatal intensive care unit admission.

**Conclusion:**

Because ICI discontinuation and ICI-T1DM carry risks for the patient and child, the decision regarding pregnancy warrants careful consideration. Diabetologists should collaborate with patients and other clinical departments to develop a treatment plan for childbirth.

## Introduction

Immune checkpoint inhibitors (ICIs) are widely applied for advanced cancers. ICIs exert their antitumor effects by inhibiting the transmission of immunosuppressive signals and deactivating the suppression of the T-cell immune response [1]. This pharmacological effect is considered harmful during pregnancy because its target molecules are expressed at the fetomaternal interface, and ICI induces disruption of immune tolerance [2]. Thus, it is recommended that patients not become pregnant for several months after ICI discontinuation. The discontinuation period varies for each ICI, and it is 5 months for nivolumab according to the European Medicines Agency (EMA) technical sheet [3].

ICI-induced type 1 diabetes (ICI-T1DM) is a rare immune-related adverse event [4] but the report of ICI-T1DM has increased over time with the increased use of ICIs [5]. We herein present the first ICI-T1DM patient, to our knowledge, who experienced delivery after advanced cancer survival with ICI therapy.

## Case description

A 31-year-old woman was diagnosed with accessory parotid cancer (T2N0M0) and underwent total parotidectomy. The pathological diagnoses were salivary duct carcinoma, which is a highly malignant salivary gland tumor. She received postoperative adjuvant therapy (radiotherapy combined with cisplatin). One year after the surgery, computed tomography revealed multiple pulmonary metastases. ICI treatment using nivolumab was initiated. The metastases disappeared after 19 cycles of nivolumab.

She had never been diagnosed with diabetes. Her random blood glucose level was lower than 100 mg/dL each time until the 42nd cycle and 144 mg/dL before the 43rd cycle (22 months after the first administration). The patient developed thirst and polydipsia 2 days after 43 cycles. Laboratory findings 6 days after 43 cycles are shown in Table 1. Her random blood glucose level was 481 mg/dL, and her HbA1c level was 6.6%. Low 24-hour urinary C-peptide and glucagon stimulation tests suggested insulin deficiency. Her body weight was of 48.2 kg (BMI 17.1). She was diagnosed with ICI-T1DM and started multiple daily insulin therapy. Complete response was maintained for 2 years by nivolumab, and she finished nivolumab in 77 cycles to attempt pregnancy.

**Table 1:** The clinical course at ICI-T1DM diagnosis

	ICI-T1DM diagnosis	References
Diabetes-related data		
HbA1c (%)	6.6	4.6–6.2
Random blood glucose (mg/dL)	481	60–110
Urinary C-peptide (μg/day)	6.5	20.5–198
GAD antibody (U/mL)	<0.5	<5.0
IA-2 antibody (U/mL)	<0.6	<0.6
ZnT8 antibody (U/mL)	<10.0	<15.0
Biochemistry		
Blood urea nitrogen	11.4	
Creatinine	0.55	
Amylase (U/L)	60	
Elastase (ng/dL)	117	< 300
Lipase (IU/L)	42	11–59
Total ketone bodies (μmol/L)	4859	< 130
3-OHBA (μmol/L)	3916	< 85
Acetoacetic acid (μmol/L)	943	< 55
TSH (μIU/mL)	1.04	0.50–5.00
FT3 (ng/dL)	2.05	2.05–4.30
FT4 ( pg/mL)	1.46	0.90–1.70
Urinalysis		
Urinary ketone	(3 + )	(—)
Urinary albumin (mg/day)	7.1	< 30
Arterial blood gas analysis		
pH	7.367	7.35–7.45
pCO2 (mmHg)	33.5	32.0–42.0
HCO3- (mmol/L)	18.8	20–24
Base excess (mmol/L)	−5.6	−3.3–2.3
Glucagon stimulation test		
C-peptide (0 min) (ng/mL)	0.06	0.80–2.50
C-peptide (6 min) (ng/mL)	0.07	
HLA haplotype		
A2	02:01	
DR3	Negative	
DR4	Negative	
DR9	Negative	

*GADA* glutamic acid decarboxylase, *IA-2* islet antigen 2, *ZnT8* zinc transporter 8, *TSH* thyroid-stimulating hormone, *FT3* free triiodothyronine, *FT4* free thyroxine

To achieve better glycemic control for pregnancy, she started sensor-augmented pump (SAP) with predictive low glucose suspend (PLGS) (Minimed^TM^ 640 G system), which was the latest automated insulin delivery (AID) system in Japan. The PLGS algorithm was set at 60 mg/dL. She had undetectable serum and urinary C-peptide when she started SAP. Her HbA1c level decreased from 7.8 to 6.6% without increasing hypoglycemia in one year. Although 5 embryos had been cryopreserved immediately after her parotid cancer diagnosis, ovum pickup and in vitro fertilization were performed again one year after ICI discontinuation due to poor-quality cryopreserved embryos. She then underwent transcervical resection of endometrial polyps. During hospitalization, she switched to the Minimed^TM^ 770 G system to improve the continuous glucose monitoring (CGM) sensor accuracy.

**Fig. 1:**
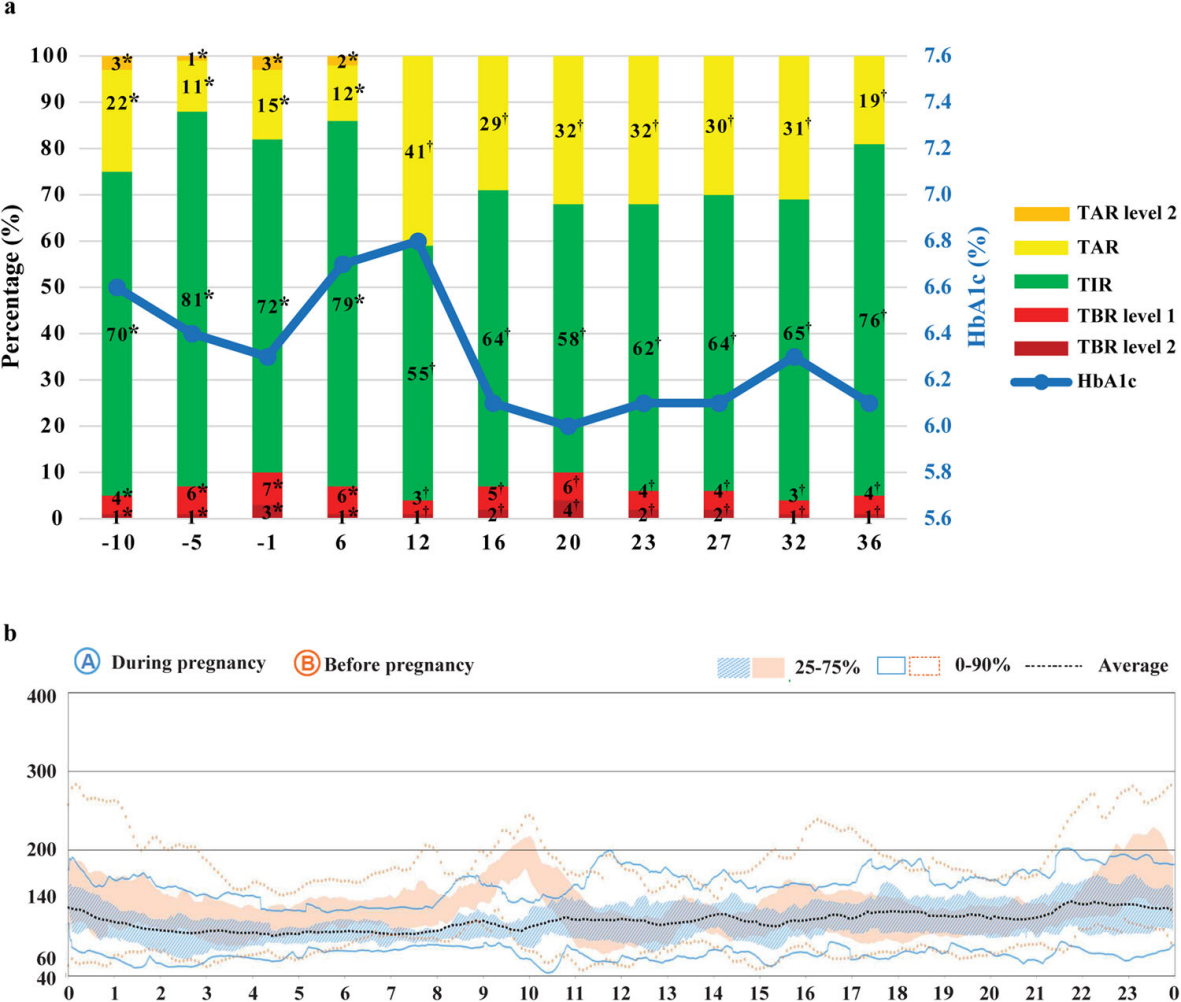
Glycemic profiles during pregnancy. **a** Continuous glucose monitoring profiles and HbA1c levels before and during pregnancy. * Before pregnancy confirmation, each CGM metric was calculated according to the CGM targets for adults with type 1 diabetes: TIR 70–180 mg/dL. TAR 181–249 mg/dL, TAR level 2 250 mg/dL, TBR level 1 54–69 mg/dL, TBR level 2 < 54 mg/dL. † After confirmation of pregnancy, each CGM metric was calculated according to the CGM targets for pregnant women with type 1 diabetes: TIR 63–140 mg/dL. TAR > 140 mg/dL, TBR level 1 54–63 mg/dL, TBR level 2 < 54 mg/dL. CGM continuous glucose monitoring, TIR time in range, TAR time above range, TBR time below range. **b** Diurnal glycemic changes before pregnancy (orange) and during pregnancy (blue)

The patient remained in complete response after ICI discontinuation, and embryo transfer was initiated 19 months after ICI discontinuation. Pregnancy was confirmed after a second embryo transfer (21 months after ICI discontinuation). Although she achieved 79% of time in range (TIR, a target range of 70–180 mg/dL) at the time of the pregnancy confirmation (Fig. 1a), her TIR calculated using the pregnancy target range (63–140 mg/dL) was 57%. Her glycemic control became slightly worse in early pregnancy due to an unexpected increase in carbon hydrate intake to reduce hyperemesis gravidarum. To decrease fasting blood glucose levels, the PLGS algorithm was altered to 50 mg/dL in two months. To minimize postprandial glycemic excursions, we decreased the carbohydrate ratio and adjusted the timing of meal bolusing. She finally achieved the CGM-based target during pregnancy (Fig. 1a, b).

At 30 weeks of gestation, her fetus was suspected of having hypospadias by ultrasonography. She did not present with hypertension or proteinuria until the examination at 35 weeks. At 36 weeks and 6 days, she was admitted to our hospital with preeclampsia. Her blood pressure was 152/84 mmHg, and urinalysis revealed proteinuria (5.34 g/g creatinine). Due to bilateral pleural effusion and dyspnea, an emergency cesarean section was performed on that day. She delivered a boy weighing 2682 g. His Apgar score was 8 at 1 min and 9 at 5 min. Although he did not require neonatal intensive care unit admission, he had hypospadias and bifid scrotum with normal testicular size. The boy showed normal development on examination one month after discharge and was scheduled to undergo phalloplasty and urethroplasty.

## Discussion

The present case highlights the development of ICI-T1DM in a woman of childbearing age and successful delivery with adequate glycemic control using the AID system and complete response after ICI discontinuation. It was achieved through the patient’s strong willingness for childbirth, adequate understanding, and continuous communication between the tumor treatment team, the obstetrics and gynecology team, the diabetes support team, and the patient. As suggested by a consistent increase in ICI-T1DM [5] and association between higher risk of ICI-T1DM development and younger age [4], there is a potential for a future increase in the number of women of childbearing age with ICI-T1DM. We hope our experience proves to be invaluable in addressing those cases.

The patient had a strong willingness to undergo childbirth and underwent embryo cryopreservation soon after the cancer diagnosis. Maintenance of complete response resulted in her putting that willingness into action. ICI discontinuation and nonactive treatment may lead to lower overall survival than active treatment [6]. Thus, it is difficult for medical providers to recommend childbirth. The decision was based on the patient’s considerable willingness and adequate understanding of the risks to both the patient and her child. Due to the lack of evidence about cancer recurrence after ICI discontinuation, the patient and her oncologist decided to have a one-year follow-up period under careful monitoring. Using this period, obstetricians and gynecologists assessed reproductive function and prepared for pregnancy. As diabetologists, we introduced SAP and improved glycemic control. These cooperations beyond clinical departments resulted in delivery without lethal complications. Although the importance of care plans for cancer survivors has been noted [7], there is no clear landmark on care plans for those treated with ICIs.

The prevalence of ICI-T1DM was less than 1%, and the average onset age was over 60 years [4, 8]. Although younger age was reported to be a risk factor for ICI-T1DM development, the absolute number of ICI-T1DM patients under 60 years old was not large probably due to the less chance to use ICIs among younger patients [4]. These results suggested the rarity of ICI-T1DM in women of childbearing age. ICI-T1DM is characterized by a high incidence of diabetic ketoacidosis and low C-peptide [8]. ICI-T1DM often shows a fulminant phenotype as observed in our case. Our facility reported four out of six cases showed a fulminant phenotype [9]. In pregnant women with T1DM, high HbA1c is associated with both maternal and neonatal complications [10]. However, precise glycemic control during pregnancy is challenging despite the use of AID systems [11]. Furthermore, patients with undetectable C-peptide showed a significantly higher M-value than those with detectable C-peptide [12], suggesting difficulty in maintaining the narrow glycemic target range during pregnancy in such patients. In this case, the patient decided to use SAP with PLGS. With adequate patient’s management, the therapy minimized postprandial glycemic excursions and hypoglycemia, which resulted in achieving glycemic targets during pregnancy.

In this case, there were maternal and neonatal complications, including preeclampsia, preterm delivery, and congenital malformations, even under relatively good glycemic control. T1DM is associated with an increased risk of preeclampsia and preterm delivery [10, 13]. Hypospadias is one of the most common urogenital malformations and occurs in 0.3% of Asian male infants [14]. Bifid scrotum was associated with 33% of hypospadias [15]. The present patient was of primiparity, high maternal age (>=35), and prepregnancy diabetes, which were risk factors for hypospadias [16, 17]. A case series summarized that eight out of nine infants exposed to ICIs were preterm, and one of them had congenital malformation [18]. This might suggest that ICI use is associated with these complications. In this case, ovum pick up was performed 15 months after ICI discontinuation and embryo transfer was initiated 19 months after ICI discontinuation. These periods are much longer than the 5 months suggested by the EMA technical sheet [3], suggesting the limited effect of ICI on these complications. Because various factors may affect pregnancy and neonatal complications as mentioned above, the precise association between the complications and previous ICI use or ICI-T1DM remains ambiguous. Further research is needed to examine the association.

In conclusion, we reported an ICI-T1DM patient who experienced delivery after advanced cancer survival with ICI therapy. While the importance of care for cancer survivors has been acknowledged, care planning for those after ICI therapy has not been established. We hope that this report will provide one of the paths forward in optimizing the care plan.
